# S100A12 Suppresses Pro-inflammatory, but Not Pro-Thrombotic Functions of Serum Amyloid A

**DOI:** 10.1371/journal.pone.0062372

**Published:** 2013-04-24

**Authors:** Yuen Ming Chung, Jesse Goyette, Nicodemus Tedla, Kenneth Hsu, Carolyn L. Geczy

**Affiliations:** 1 Inflammation and Infection Research Centre, University of New South Wales, Sydney, New South Wales, Australia; Cardiff University School of Medicine, United Kingdom

## Abstract

S100A12 is elevated in the circulation in patients with chronic inflammatory diseases and recent studies indicate pleiotropic functions. Serum amyloid A induces monocyte cytokines and tissue factor. S100A12 did not stimulate IL-6, IL-8, IL-1β or TNF-α production by human peripheral blood mononuclear cells but low amounts consistently reduced cytokine mRNA and protein levels induced by serum amyloid A, by ∼49% and ∼46%, respectively. However, S100A12 did not affect serum amyloid A-induced monocyte tissue factor. In marked contrast, LPS-induced cytokines or tissue factor were not suppressed by S100A12. S100A12 did not alter cytokine mRNA stability or the cytokine secretory pathway. S100A12 and serum amyloid A did not appear to form complexes and although they may have common receptors, suppression was unlikely via receptor competition. Serum amyloid A induces cytokines via activation of NF-κB and the MAPK pathways. S100A12 reduced serum amyloid A-, but not LPS-induced ERK1/2 phosphorylation to baseline. It did not affect JNK or p38 phosphorylation or the NF-κB pathway. Reduction in ERK1/2 phosphorylation by S100A12 was unlikely due to changes in intracellular reactive oxygen species, Ca^2+^ flux or to recruitment of phosphatases. We suggest that S100A12 may modulate sterile inflammation by blunting pro-inflammatory properties of lipid-poor serum amyloid A deposited in chronic lesions where both proteins are elevated as a consequence of macrophage activation.

## Introduction

Serum amyloid A (SAA) is an acute-phase reactant principally produced in response to injury, infection and inflammation [1]. The liver is the primary site of synthesis, although other cell types, including normal epithelial cells, extravascular lymphocytes and plasma cells, and endothelial cells [2] can express SAA; activation by pro-inflammatory cytokines can induce its expression in monocytes/macrophages [3], THP-1 monocytoid cells [4], smooth muscle cells (SMC) and endothelial cells [3]. Elevated levels of SAA are found in patients with infections [5], and clinical studies associate changes in SAA levels with progress of chronic inflammatory diseases with inflammatory components such as diabetes [6], cardiovascular disease [7], rheumatoid arthritis [8] and neoplasia [9]. SAA3 is primarily associated with high density lipoprotein (HDL) in the circulation [10], [11] but is also deposited in inflammatory lesions [12], [13]. In atheroma, it is seen in endothelial cells, SMC, macrophage-derived “foam cells”, adventitial macrophages and adipocytes [3] and SAA overexpression in apolipoprotein (Apo) E^−/−^ mice increased plasma levels of interleukin (IL)-6, tumour necrosis factor-α (TNF-α) and chemokine (C–C motif) ligand-2 and accelerated progression of atherosclerosis [14].

Since our initial studies describing cytokine [15] and tissue factor (TF) [16] induction by SAA-activated monocytes/macrophages, together with its ability to promote endothelial cell dysfunction [17], [18], there is increasing interest in mechanisms relating to SAA’s pro-inflammatory function. SAA induces pro-inflammatory cytokines (eg. IL-1β, IL-6, IL-8, TNF-α, and interferon-γ) in neutrophils [19], [20], monocytes [15], [21] and lymphocytes [22], and is a leukocyte chemoattractant [23], [24]. Several receptors are implicated, including the receptor for advanced glycation end products (RAGE) [16], [25], formyl peptide receptor-like (FPRL)-1 and -2 [20], [26]–[29], toll-like receptor (TLR)-2 and -4 [30]–[32], and scavenger receptors CLA-1/SR-B1 [33]–[35] and CD36 [36] that modulate innate immune responses to several ligands. Recent studies suggest that in macrophages, four signaling pathways involving nuclear factor-κB (NF-κB) and three mitogen-activated protein kinase (MAPK) may contribute to cytokine production (summarized in [36]).

S100A12, S100A8 and S100A9 (collectively known as calgranulins), are a subset of S100 Ca^2+^-binding proteins elevated in serum from patients with various inflammatory conditions [37]. S100A12 is constitutively expressed in neutrophils (∼5% of cytosolic protein) [38] and is inducible in peripheral blood monocytes by lipopolysaccharide (LPS) and TNF-α [39], and in human macrophages by IL-6 [40]. S100A12 is present in foam cells and macrophages in atherosclerotic lesions [41], in neutrophils in rheumatoid synovial lining [39], in eosinophils and macrophages in airway tissue from asthmatic lungs [42], and in infiltrating neutrophils and macrophages in chronic inflammatory bowel disease [43], [44]. High circulating levels of S100A12 are present in sera from patients with chronic inflammatory diseases including atherosclerosis [41], rheumatoid arthritis [45] and Kawasaki disease [46].

Pro-inflammatory functions for SAA and S100A12 are reported [39], [47], [48], and they may share common receptors and signal transduction pathways, such as via RAGE and/or a pertussis toxin-sensitive G-protein-coupled receptor (G-PCR) [47], [49]. Interactions of SAA with RAGE [25] and with CD36 [36] are implicated in cytokine induction. SAA induction of TF is partially mediated by RAGE on monocytes [16], and on endothelial cells via FPRL-1 [29], a human G-PCR with low affinity for N*-*formyl peptides; the p38 and extracellular signal-regulated kinases 1/2 (ERK1/2) MAPK, and NF-κB pathways are implicated in activation [16].

Functions first described for S100A12 suggested a pro-inflammatory role, although recent studies indicate pleiotropic activities [41], [50]. S100A12 at low concentrations is chemotactic for neutrophils, monocytes and mast cells [42], [49]; it provokes mast cell activation and leukocyte recruitment *in vivo*, possibly involving a G-PCR [39], [49]. S100A12 does not induce matrix metalloproteinase (MMP) genes in human macrophages, but profoundly inhibits MMP-2, -3, and -9 activities via Zn^2+^ chelation, and may modulate MMP activities in inflammatory lesions [41].

Here we describe a novel regulatory role for S100A12 by downregulating SAA’s ability to induce pro-inflammatory cytokines. S100A12 did not promote cytokine production by peripheral blood mononuclear cells (PBMC), but significantly inhibited SAA-induced IL-6 and TNF-α mRNA levels, and IL-8 and TNF-α production by human PBMC, whereas LPS-induced responses were not affected. In contrast, S100A8 and S100A9 were not significantly suppressive. Interestingly, S100A12 did not influence monocyte TF induction by SAA or by LPS. Cytokine induction at the SAA concentrations tested did not promote a Ca^2+^ influx or generate reactive oxygen species (ROS) as reported in other studies [17], [51], [52]. Inhibition of cytokine induction was via the ERK1/2 MAPK pathway; p38 and c-Jun N-terminal kinase (JNK) MAPK, or the NF-κB transcription pathways were unaffected. These studies suggest that S100A12 may protect monocytes/macrophages from activation by SAA during inflammatory episodes.

## Materials and Methods

### Reagents

Human recombinant Apo-SAA (a consensus protein corresponding to human Apo-SAA1α) was from PeproTech Inc. (Rocky Hill, NJ), human recombinant ApoD from Sigma Aldrich (St. Louis, MO) and human recombinant ApoE3 from ProSci Inc. (Poway, CA); all had endotoxin content of <0.1 ng/µg protein. Human recombinant S100A8, S100A9 [53] and S100A12 [39] were produced in-house using the pGEX-2T expression system, purified using reverse phase-high performance liquid chromatography and maintained under argon gas to prevent oxidation. To generate the S100A8/S100A9 complex, equimolar amounts (0.5 nM) of recombinant S100A8 and S100A9 were incubated together in Ca^2+^-containing RPMI for 20 min at room temperature (RT) before use. TRIzol reagent and SuperScript VILO cDNA Synthesis Kit were from Invitrogen, Life Technologies (Carlsbad, CA) and co-precipitant from Bioline (London, UK). TURBO-DNase was from Ambion (Austin, TX), LightCycler 480 SYBR Green I Master from Roche (Mannheim, Germany); other reagents for real-time RT-PCR were from Invitrogen, Life Technologies. Rabbit anti-human IκBα, p44/42 MAPK (ERK1/2), phospho-p44/42 MAPK, phospho-MEK1/2 (Ser_217/221_), MEK1/2, p38, phospho-p38, SAPK/JNK, phospho-Ca^2+^/calmodulin (CaM)-dependent protein kinases II (CaMKII) (Thr_286_) antibodies (Ab) were from Cell Signaling Technology (Danvers, MA). Rabbit anti-active JNK Ab was from Promega (Madison, WI). FITC-conjugated anti-human IL-8 and control human IgG were from BD Biosciences (San Jose, CA). Rabbit anti-human NF-κB p65 (C-20) was from Santa Cruz Biotechnology (Santa Cruz, CA). HRP-conjugated goat anti-rabbit or goat anti-mouse IgG were from Bio-Rad Laboratories Inc. (Hercules, CA). Goat anti-rabbit IgG-Alexa-Fluor-568 was from Molecular Probes, Life Technologies and mouse anti-human Src homology region 2 domain-containing phosphatase-1 (SHP-1) Ab was from Millipore (Billerica, MA).

Culture media for all experiments were sterilized by filtration through 0.22 µm Zetapore membranes (Cuno, New South Wales, AUS) to remove contaminating LPS. Media, cell culture reagents, and recombinant S100 proteins were routinely monitored and only used if endotoxin levels were <20 pg/ml (chromogenic *limulus* amoebocyte lysate assay; Associates of Cape Cod, East Falmouth, MA).

### Mononuclear Cell Culture and Stimulation

PBMC isolated from blood of healthy subjects [54] by density-gradient centrifugation using Ficoll-Paque Plus (GE Healthcare Life Sciences; Buckinghamshire, UK) were washed three times with Ca^2+^-free HBSS (Sigma). Cell numbers were analyzed using a Beckman Coulter Counter and generally contained ∼10% monocytes, 90% lymphocytes and <1.5% granulocytes. PBMC (1.5–2.0×10^6^/well) in serum-free RPMI 1640+100 U/ml penicillin, 100 µg/ml streptomycin and 2 mM L-glutamine (GIBCO, Life Technologies) were dispensed into 24-well NUNC plates (Thermo Fisher Scientific, Waltham, MA) and incubated with the appropriate stimulants for the indicated times at 37°C in 5% CO_2_ in air.

Monocytoid THP-1 cells (American Type Tissue Culture Collection, Manassas, VA; TIB-202) were maintained in RPMI 1640 supplemented with 10% heated (56°C, 30 min) FBS, 2-ME (50 µM), 100 U/ml penicillin, 100 µg/ml streptomycin and 2 mM L-glutamine at ∼2.0–2.5×10^5^/ml. For stimulation, THP-1 cells (0.5×10^6^/well) were seeded into 24-well NUNC plates in serum-free RPMI 1640 with appropriate stimulants for the indicated times, at 37°C in 5% CO_2_ in air.

### Quantitative Real-time RT-PCR for Cytokines, TF and NF-κB1

After stimulating for the times indicated, cells were lysed with TRIzol reagent and RNA prepared as described [55]. RNA (1.5 µg) was treated with Turbo DNase and reverse transcribed using SuperScript VILO cDNA synthesis kit according to manufacturer’s instructions. Negative controls (no first-strand synthesis and no template control) were prepared by performing reverse transcription reactions in the absence of Superscript Enzyme Mix and cDNA.

PCR amplification for IL-6, IL-8, IL-10, TNF-α, TF, NF-κB1 and β-actin was performed with LightCycler 480 SYBR Green I Master Mix as described [15], [41], [56]. Assays performed in duplicate containing 5 µl 2x SYBR Green I Master Mix, 4 µl template cDNA or negative control, 1 µl 2.5 µM forward and reverse combined primers (primer sequences were as listed in [15], [41], [56] in a final volume of 10 µl, and analyzed in 384-multiwell optical reaction plates (Roche). Reactions were amplified and quantified using the LightCycler 480 system (Roche) with standard cycle conditions, and analyzed using the appropriate software. Relative quantities of mRNA in duplicate samples were calculated by the comparative cycle threshold (C_T_) method and normalized against human β-actin mRNA as endogenous control. In addition to β-actin, real-time RT-PCR analysis of cytokine suppression by S100A12 and stability of IL-6 mRNA were normalized to HPRT as housekeeping gene and results were no different to those obtained when normalized against β-actin.

To determine whether S100A12 suppression of cytokine levels was due to mRNA stability, the half-life of cytokine mRNA was measured by culturing THP-1 cells with S100A12, SAA or both for 4 h at 37°C in 5% CO_2_ in air. Actinomycin D (10 µg/ml; Sigma) was subsequently added to block transcription, and cells immediately returned to 37°C. Cells were harvested immediately or following 30, 60, 90, 120 and 180 min. Levels of IL-6 and TNF-α mRNA were determined as described above.

### Cytokine Measurement

Culture supernates from stimulated PBMC and THP-1 cells were assayed in duplicate for IL-6, IL-8, TNF-α and IL-1β levels using cytokine-specific DuoSet ELISA kits (R&D Systems; Minneapolis, MN) according to manufacturer’s instructions.

Intracellular IL-8 levels in SAA ± S100A12-treated THP-1 cells were determined by flow cytometry. Stimulated THP-1 cells (2×10^5^) were transferred to FACS polystyrene tubes (Becton Dickinson (BD); Franklin Lakes, NJ), washed with cold PBS containing 0.5% BSA and 0.1% sodium azide, pre-fixed with 4% paraformaldehyde (400 µl) for 10 min at RT, washed with 10x volumes cold wash buffer, then permeabilized with PBS containing 0.1% saponin and 1% BSA (100 µl) for 15 min at RT, with shaking. Cell suspensions (50 µl) were transferred to FACS tubes, incubated with FITC-conjugated anti-human IL-8 (3 µg/ml) or control human IgG (3 µg/ml), vortexed, incubated for 30 min at RT in the dark, then washed and fixed in 1% paraformaldehyde in PBS, and analyzed by flow cytometry (FACSCalibur; BD Biosciences). In all experiments, 10,000 events were collected from a large gate to exclude debris, but to include all cells.

### SDS-PAGE and Western Blotting

To determine whether SAA and S100A12 formed complexes that may affect SAA function, S100A12 (1 µg) was cross-linked with SAA (1 µg) using bis[sulfosuccinimidyl] suberate (Pierce, Rockford, IL; 5 µM) in the presence or absence of 1 mM Ca^2+^. Proteins were suspended in PBS and cross-linked for 30 min at RT in the dark, according to manufacturer’s instructions. Complexes were resolved on 10% SDS-PAGE gels under non-reducing conditions, then silver stained as described [57].

To assess whether NF-κB and MAPK (ERK1/2, MEK1/2, p38 and JNK), CaMKII or SHP-1 signaling pathways were involved in S100A12 suppression of SAA-mediated cytokine production, time dependent IκB degradation and phosphorylation state of ERK1/2, MEK1/2, p38, JNK, CaMKII and SHP-1 were detected by Western blotting [25], [30]. After stimulation for the appropriate time, cells were washed once with cold PBS, then lysed in lysis buffer containing 50 mM Tris; pH 7.5, 150 mM NaCl, 1 mM EDTA, 1 mM EGTA, 20 mM NaF, 20 mM Na_4_P_2_O_7_, 2 mM Na_3_VO_4_, 10% glycerol, 1% NP-40, 0.1% SDS, 0.5% deoxycholate, 1 mM PMSF, and Complete Protease Inhibitor Cocktail tablet (Roche) (one tablet/50 ml solution) for 15 min. Detergent-insoluble materials were pelleted at 12,000×*g* for 15 min at 4°C and supernatant removed. Total protein levels were quantitated using the BCA Protein Assay Kit (Thermo Fisher Scientific). Protein samples (30 µg) were separated by 10% SDS-PAGE, transferred to a 0.2 µm polyvinylidene difluoride membrane and immunoblotted with specific antibodies (1∶1000 v/v) at 4°C O/N. Reactivity was detected with HRP-conjugated goat anti-rabbit (1∶3000 v/v) or goat anti-mouse (1∶2000 v/v) IgG for 1 h at RT, followed by 3×5-min washes, and reactivity visualized by Western Lightning-enhance chemiluminescence substrate.

Immunofluorescence for NF-κB p65 nuclear translocation in THP-1 cells treated with SAA ± S100A12 was performed as described [25].

### Para-nitrophenyl Phosphate (pNPP) Phosphatase Assay

The general phosphorylation activity of SAA ± S100A12-treated THP-1 cells was measured by assessing the total phosphatase activity using pNPP as a substrate [58]. Stimulated cells (10^6^) were collected, washed once with PBS, then lysed with 250 µl reaction mixture containing 1.5 mM EDTA, 37.5 mM Na acetate, 0.15% w/v Triton X-100, 3% w/v glycerol and 5 mM DTT. For kinetic reactions, 100 µl pNPP (6.3 mg/ml dissolved in 0.1 M glycine, pH 10.4 containing 1 mM MgCl_2_ and 1 mM ZnCl_2_) was mixed with reaction mixture (100 µl). Samples were incubated at 37°C for 30 min, then quenched with 50 µl 3 M Tris. Release of para-nitrophenyl was determined spectrophotometrically by measuring A_405 nm_, and absorbance calculated as a ratio of enzyme activity relative to control.

### Intracellular ROS Production and Ca^2+^ Measurement

To assess whether SAA and/or S100A12 altered intracellular ROS production in PBMC, we measured its levels by incubating cells with CM-H_2_DCFDA (5-(and-6)-chloromethyl-2′,7′-dichlorodihydrofluorescein diacetate, acetyl ester; Molecular Probes, Life Technologies), based on the manufacturer’s protocol. CM-H_2_DCFDA is hydrolyzed intracellularly by esterases to H_2_DCF, a non-fluorescent and membrane-impermeable product, and subsequently oxidized to fluorescent DCF in the presence of ROS.

PBMC (2×10^5^ monocytes/treatment) were incubated for 30 min with 5 µM CM-H_2_DCFDA in HBSS containing Ca^2+^/Mg^2+^, supplemented with 0.1% BSA and 10 mM HEPES at 37°C in 5% CO_2_ in air, then washed twice and incubated with the appropriate stimulus for 30 min. DCF fluorescence (Ex = 485 nm; Em = 530 nm) was analyzed by flow cytometry on cells gated on forward/side scatter profiles for the monocyte population; 5000 events were collected from a large gate that excluded debris, but included all cells.

A second approach to investigate whether SAA altered intracellular ROS levels in monocytes, was to use diphenyleneiodonium (DPI), an inhibitor of NADPH oxidase, to reduce ROS generation [59]. THP-1 cells were treated with SAA (1 nM) ± DPI (5 µM; Sigma), and IL-8 levels measured by ELISA.

Ca^2+^ flux was determined with the Ca^2+^-sensitive fluorochrome (Fluo-3/acetomethyl ester) (Invitrogen, Life Technologies) using a PerkinElmer LS 55 fluorescence spectrophotometer (Waltham, MA) as described [17] using THP-1 cells. To measure [Ca^2+^]_i_, cells in 2 ml were placed in a quartz cuvette with a magnetic flea in a Perkin-Elmer LS 55 spectrofluorimeter (Perkin-Elmer, Waltham, MA, USA) and stimulated with various concentrations of SAA (5 µl aliquots). Ionomycin (1 µg/ml) was used as a positive control to achieve maximum Ca^2+^ flux; EGTA (5 mM) was used to inhibit cells to estimate an approximate minimal response. Fluorescence was measured at 506 nm (excitation wavelength) and 526 nm (emission wavelength) every 100 msec. Results expressed as relative fluorescence units at 506 nm over time.

Superoxide dismutase (SOD) levels in S100A12± SAA-treated THP-1 cells were determined based on inhibition of pyrogallol, using a 96-well microassay as described in [60].

### Assessment of Apoptosis/viability

The viability of stimulated THP-1 cells was evaluated using the Annexin V-PE apoptosis detection kit I (BD Pharmingen) following manufacturer’s instructions. Briefly, THP-1 cells (2.5×10^5^ in 24-wells NUNC plates) were stimulated with SAA, S100A12 or both for ∼15 h at 37°C in 5% CO_2_ in air, then washed twice with cold PBS, and resuspended in Annexin V binding buffer (10 mM Hepes/NaOH, pH 7.4 with 140 mM NaCl and 2.5 mM CaCl_2_; 1×10^6^ cells/ml). Cell suspensions (1×10^5^/100 µl) were transferred to FACS polystyrene tubes, 5 µl Annexin V-PE and 5 µl 7-AAD added, and cells incubated for 15 min at RT in the dark, with gentle shaking. Annexin V binding buffer (400 µl) was added and cells analyzed by flow cytometry immediately after staining. In all experiments, 10,000 events were collected from a large gate to exclude debris, but to include all cells.

### Measurement of TF Procoagulant Activity (PCA)

PBMC (5×10^5^/well) were cultured in 250 µl serum-free RPMI 1640± stimulants, in 96-well plates (Greiner Bio-One, Kremsmünster, Austria) in 5% CO_2_ in air, as described [16]. After stimulation for the times indicated, plates were centrifuged (1400 rpm, 10 min), supernatants discarded, cells resuspended in 250 µl RPMI 1640, then subjected to two cycles of freeze (−80°C) and rapid thawing (37°C). Cell-surface TF activity of intact, viable PBMC was measured as described in [61]. Cells were stimulated for the indicated times in Nunc-minisorp tubes (Thermo Fisher Scientific), to which monocytes do not adhere, and activity directly measured. PCA was measured using a one-stage plasma recalcification test, using an automatic coagulometer (Diagnostica Stago, France) [16] and activity calculated from a standard curve using dilutions of rabbit brain thromboplastin (Sigma-Aldrich), and expressed as mU TF/10^6^ cells.

### Statistical Analysis

Values in figures are expressed as means ± SEM. Normal distribution of data was tested and passed the D’Agostino-Pearson (Omnibus K2) normality test. Statistical analyses were performed using a paired t-test or a one-way ANOVA with Bonferroni’s correction for multiple comparisons between groups as indicated.

## Results

### S100A12 Suppressed SAA-induced Cytokine Production by PBMC

**Figure 1:**
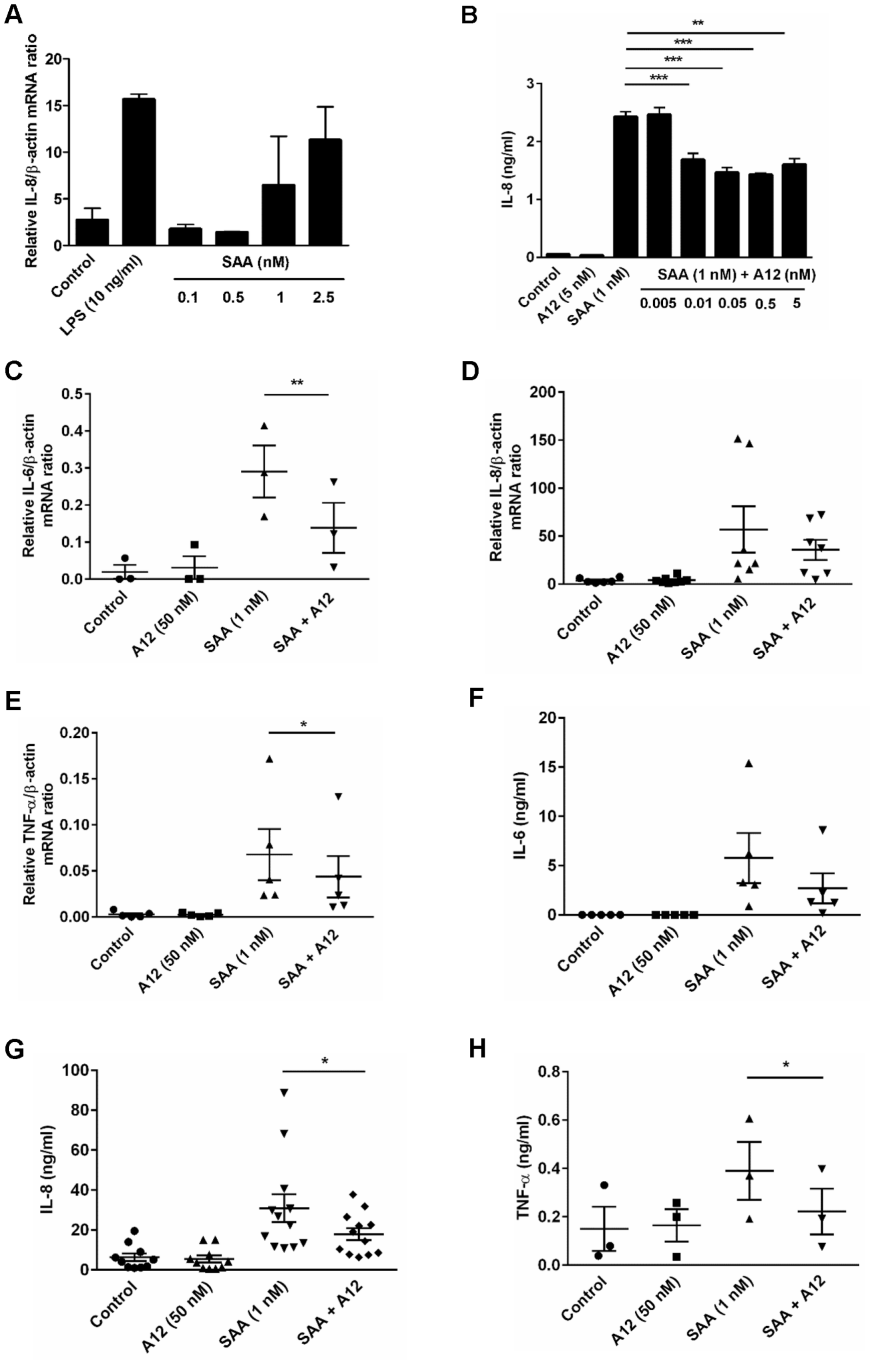
S100A12 reduced SAA-induced cytokine responses. (**A**) Dose-dependent induction of IL-8 mRNA by SAA. PBMC treated with LPS (10 ng/ml) or SAA (0.1, 0.5, 1, 2.5 nM) for 4 h, and IL-8 mRNA quantitated by real-time RT-PCR; β-actin mRNA served as endogenous control. Data represent means (relative to β-actin mRNA) ± SEM of duplicate measurements from two separate experiments. (**B**) THP-1 cells treated with SAA (1 nM), S100A12 (5 nM) and SAA (1 nM)+S100A12 (0.005, 0.01, 0.05, 0.5, 5 nM) for 15 h and IL-8 levels in supernates quantitated by ELISA. Data represent means ± SEM of duplicate measurements from three separate experiments. **p<0.01 and ***p<0.005 compared to SAA alone (analyzed by one-way ANOVA with Bonferroni’s correction for multiple comparison tests). (**C–H**) PBMC treated with SAA (1 nM), S100A12 (50 nM) or SAA ± S100A12 for 4 h (mRNA) or 15 h (protein). mRNA levels of (**C**) IL-6, (**D**) IL-8, and (**E**) TNF-α quantitated. Data represent ratios of cytokine mRNA levels relative to β-actin mRNA; means ± SEM of PBMC preparations from at least three independent donors, *p<0.05 and **p<0.01 compared to SAA alone (analyzed by paired t-tests). Levels of (**F**) IL-6, (**G**) IL-8 and (**H**) TNF-α from supernates of PBMC harvested 15 h post-stimulation quantitated by ELISA. Data represent means ± SEM of duplicate assays from at least three independent healthy blood donors, *p<0.05 compared to SAA alone (analyzed by paired t-tests).

SAA1 and SAA2 are acute-phase SAAs implicated in monocyte activation [15]. S100A12 was also implicated in leukocyte activation and cytokine generation [47], although our initial results did not reproduce these findings [41]. We next considered whether it modified functions of other PAMPs/DAMPs such as LPS or SAA. The SAA concentration (using recombinant Apo-SAA corresponding to human Apo-SAA1α) required for cytokine induction was established using PBMC because lymphocytes contribute to optimal TF induction on monocytes by SAA [62]. Since SAA associates with HDL [11], experiments were routinely carried out in lipid- and serum-free conditions in order to minimize SAA binding to serum proteins. As expected [15], PBMC cultured in media ± serum produced higher cytokine levels in serum-free conditions (not shown). IL-6 (not shown) and IL-8 mRNA (Fig. 1A) increased with 1 and 2.5 nM SAA; the suboptimal dose (1 nM) was used in subsequent experiments to allow assessment of positive or negative effects of S100A12.

As reported by us [41], we found no direct induction of pro-inflammatory cytokines by S100A12 (Fig. 1B). In contrast, S100A12 reduced IL-8 production by SAA-activated PBMC; significant suppression was seen with 50 nM S100A12 (not shown). As little as 0.01 nM S100A12 significantly suppressed IL-8 production by SAA-stimulated THP-1 cells (***p<0.005 compared to SAA-stimulated IL-8 levels; Fig. 1B), a concentration 100-fold less than the SAA used. Inhibition reached ∼38% with 0.05, 0.5 and 5 nM S100A12 (***p<0.005 and **p<0.01 compared to SAA-stimulated THP-1 cells), although increasing amounts of S100A12 caused no further suppression, suggesting multiple pathways of activation.

To confirm the effect of S100A12 on SAA-induced cytokine production, IL-8, IL-6 and TNF-α mRNA levels were quantitated. As expected [15], SAA increased cytokine mRNA and protein levels (Figs. 1C, D, E, F, G and H). S100A12 reduced SAA-induced IL-6 (Fig. 1C), IL-8 (Fig. 1D), and TNF-α (Fig. 1E) gene expression, and differences were statistically significant (IL-6 mRNA, **p = 0.0027 and TNF-α mRNA, *p = 0.0366), except for IL-8 mRNA (*p* = 0.2122). Consistently, IL-6 (Fig. 1F; *p* = 0.0635), IL-8 (Fig. 1G; *p = 0.0361) and TNF-α (Fig. 1H; *p = 0.0256) levels in supernatants from S100A12+ SAA-stimulated cells harvested 15 h post-stimulation were ∼53, 42 and 43% less than in supernatants from SAA-stimulated cells, respectively.

**Figure 2:**
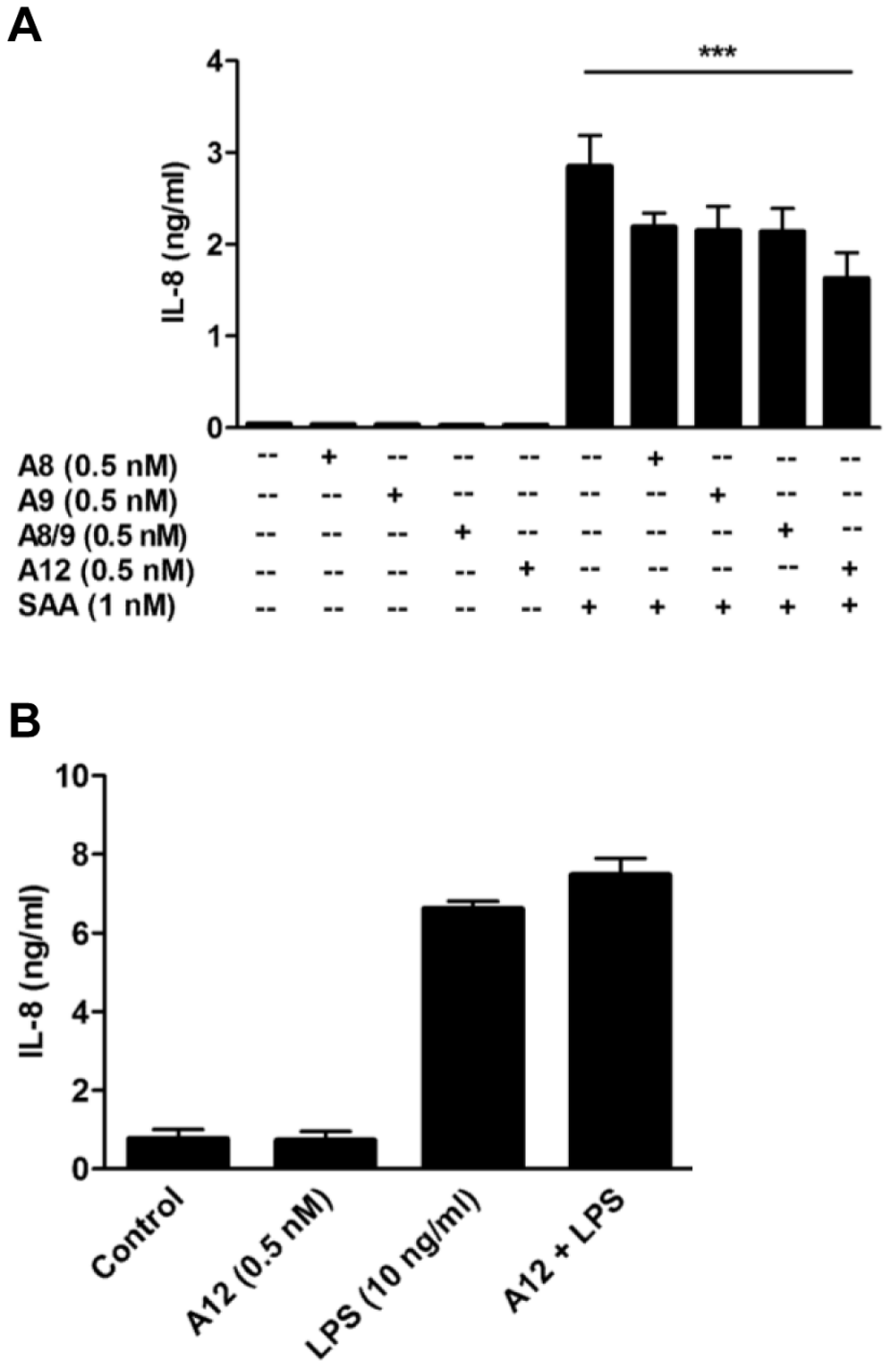
Suppression of SAA-induced IL-8 was specific to S100A12, and S100A12 did not reduce LPS-induced IL-8. (**A**) THP-1 cells incubated with SAA (1 nM) ± S100A8, S100A9, S100A8/S100A9 complex or S100A12 (0.5 nM) for 15 h, and IL-8 levels detected by ELISA. Data are means ± SEM of duplicate assays from four separate experiments; ***p<0.005 compared to SAA alone (analyzed by one-way ANOVA with Bonferroni’s correction for multiple comparison tests). (**B**) THP-1 cells treated with S100A12 (0.5 nM), LPS (10 ng/ml) or LPS+S100A12 for 15 h and IL-8 levels in supernates quantitated. Values are means ± SEM of duplicate assays from two independent experiments.

We next examined whether the inflammation-associated S100 proteins, S100A8, S100A9, or the S100A8/S100A9 calprotectin complex, which share relatively high structural homology with S100A12 [63] similarly affected cytokine induction by SAA. Although S100A8 [64] and S100A9 [65] are reported to be TLR-4 ligands, S100A8, S100A9 or the complex did not directly alter basal IL-8 levels (Fig. 2A). This contrasts with studies using S100A8 (∼0.1 µM–1 µM) to induce TNF-α, IL-1β and IL-12p70 on murine bone-marrow cells and human monocytes [66]. Sunahori *et al.* also showed induction of TNF-α, IL-1β, IL-6 and IL-8 by S100A8, S100A9 and the heterocomplex (0.1–10 µM) in human monocytes and macrophages [67]. However, our on-going studies with murine and human macrophages confirm lack of induction of numerous pro-inflammatory cytokines, and of TF, by these proteins at concentrations between 1 nM–10 µM (Hsu & Geczy, unpublished observations). When used at 0.5 nM, the amount of S100A12 that maximally suppressed IL-8 production from THP-1 cells (Fig. 1B), S100A8, S100A9, and S100A8/A9 slightly reduced IL-8, but levels were not significantly different to those induced by SAA alone. In contrast, S100A12 significantly reduced IL-8 production (***p<0.005 compared to SAA alone) (Fig. 2A).

In marked contrast to its ability to suppress SAA, S100A12 did not alter IL-8 levels induced by LPS (Fig. 2B), indicating that TLR-4-mediated signaling [68], which is also implicated in SAA signaling [32], [69], was not directly affected by S100A12.

### S100A12 did not Affect SAA-induced TF Expression of Function

**Figure 3:**
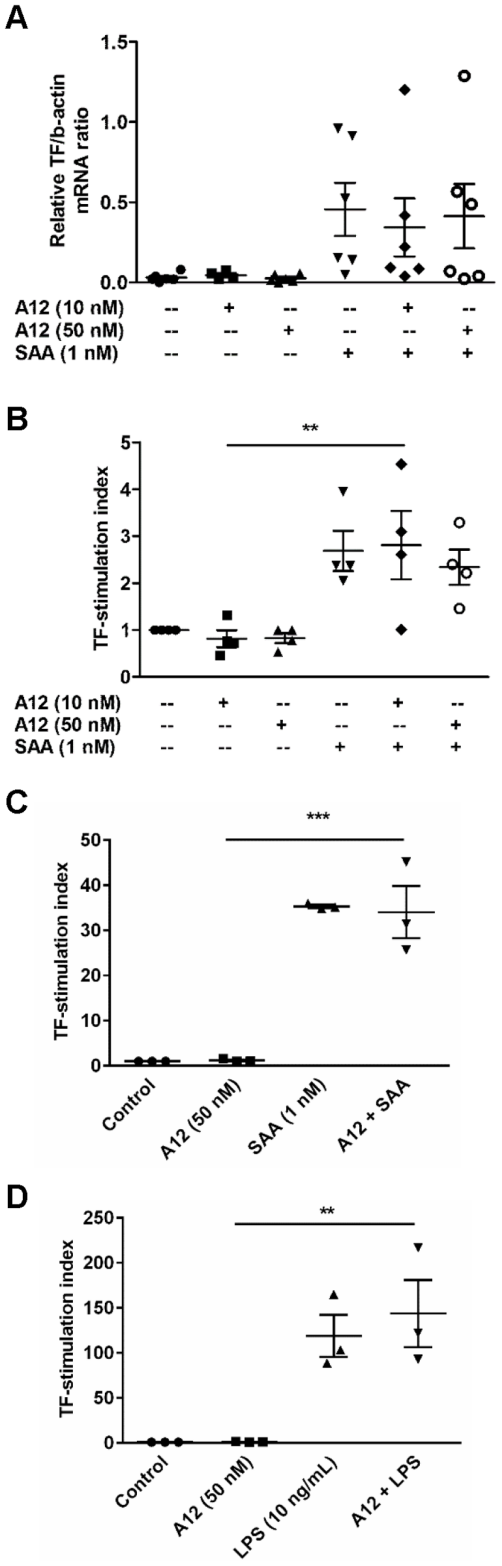
S100A12 did not suppress SAA-induced TF. (**A**) PBMC treated with SAA (1 nM), S100A12 (10 or 50 nM), or S100A12+ SAA for 2 h, and mRNA levels quantitated relative to β-actin mRNA; means ± SEM of duplicate measurements of PBMC from six independent donors (analyzed by one-way ANOVA with Bonferroni’s correction for multiple comparison tests). (**B**) PBMC stimulated as in (**A**) for 5 h, and PCA of cell lysates measured. Data expressed as TF-stimulation index (ratio of PCA of stimulated cells divided by PCA of unstimulated cells); values are means ± SEM mU TF activity assessed as recalcification time of duplicate assays using PBMC from four individual donors. **p<0.01 compared to S100A12 (10 nM)-stimulated cells (analyzed by one-way ANOVA with Bonferroni’s correction for multiple comparison tests). To determine effects of S100A12 on SAA or LPS-induced extracellular TF activity, PBMC were treated with (**C**) SAA (1 nM) ± S100A12 (50 nM) or (**D**) LPS (10 ng/ml) ± S100A12 (50 nM) in NUNC-minisorp tubes for 5 h and PCA of whole cells measured. Data expressed as TF-stimulation indices; values are means ± SEM mU TF activity assessed as recalcification time of duplicate assays using PBMC from three individual donors. **p<0.01 and ***p<0.005 compared to S100A12 (50 nM)-stimulated cells (analyzed by one-way ANOVA with Bonferroni’s correction for multiple comparison tests).

As shown by us [16], SAA induced monocyte TF mRNA (Fig. 3A) and activity, measured as recalcification time (PCA) (Fig. 3B). In marked contrast to its effects on cytokine generation, S100A12 (10 or 50 nM) did not alter basal, or SAA-induced TF mRNA (Fig. 3A). These results were reflected in the total PCA activity of lysed cells (Fig. 3B). Surface TF activity of viable cells stimulated under non-adherent conditions [61] was similarly unaffected (Fig. 3C). Similarly, S100A12 did not reduce LPS-induced TF activity (Fig. 3D).

### Mechanisms Involved in Cytokine Suppression by S100A12

**Figure 4:**
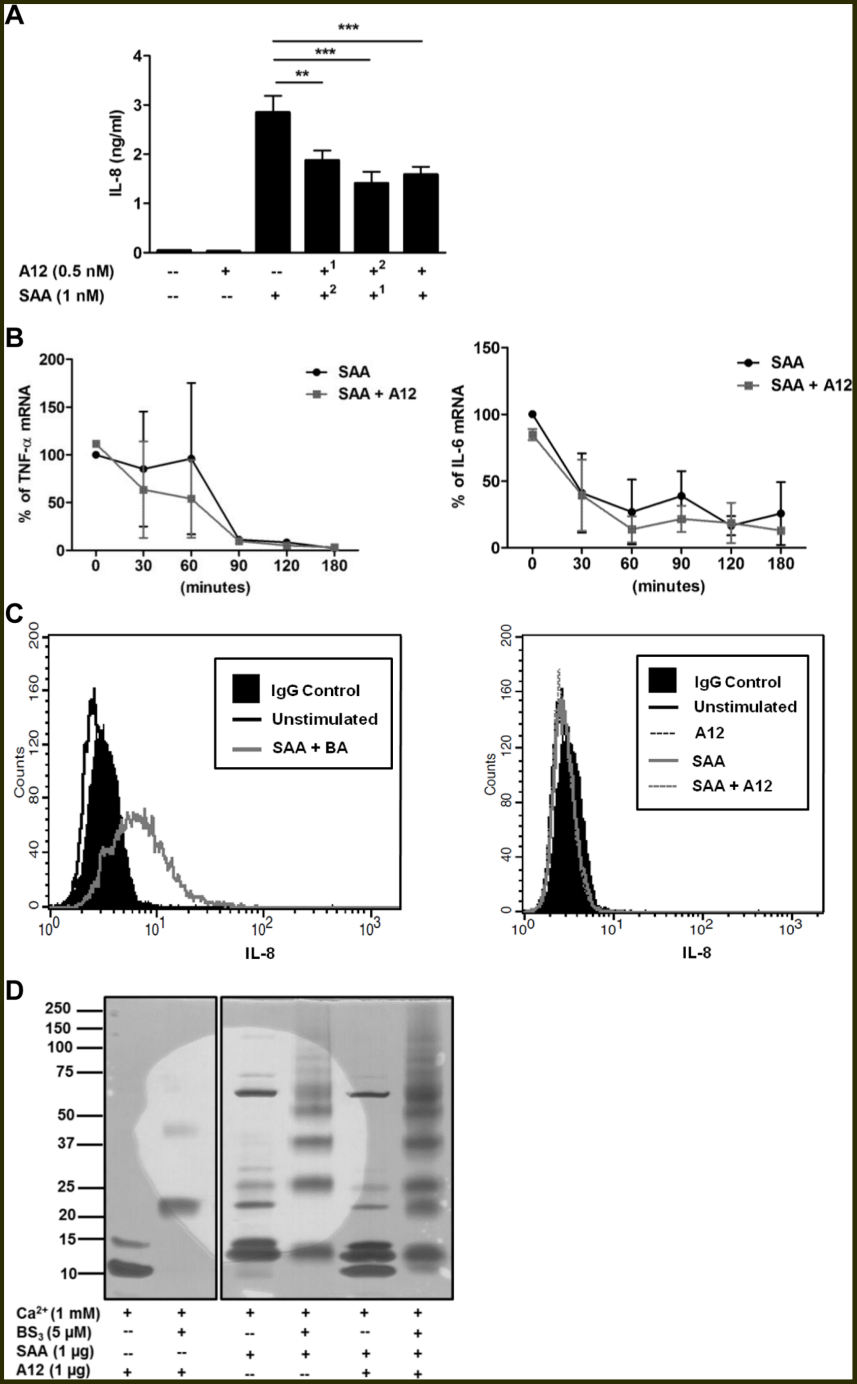
S100A12 suppression was not due to receptor competition, cytokine mRNA stability or defective IL-8 secretion. (**A**) To determine whether S100A12 or SAA competed for putative shared receptors, THP-1 cells were pre-incubated with S100A12^1^ or SAA^1^ for 30 min (where indicated) before addition of SAA^2^ or S100A12^2^ (where indicated), respectively, then cultures incubated for 15 h; IL-8 levels detected by ELISA. Data expressed as means ± SEM of duplicate assays from at least four separate experiments. **p<0.01 and ***p<0.005 compared to SAA alone (analyzed by one-way ANOVA with Bonferroni’s correction for multiple comparison tests). (**B**) Half-lives of **(left panel)** TNF-α and **(right panel)** IL-6 mRNA in THP-1 cells stimulated with SAA (1 nM) ± S100A12 (0.5 nM) determined by real-time RT-PCR. Data represents % TNF-α and IL-6 mRNA levels relative to β-actin mRNA levels; means ± SEM of duplicate assays from two independent experiments are shown. (**C**) Histograms from flow cytometric analysis of intracellular IL-8 levels in THP-1 cells. Solid black profiles represent IgG control; the black, grey or broken lines represent intracellular IL-8 levels measured 15 h post-stimulation. **(C, left panel)** THP-1 cells treated with SAA (1 nM)+brefeldin A (5 ng/ml) was the positive control; BA, brefeldin A. **(C, right panel)** THP-1 cells treated with SAA (1 nM), S100A12 (0.5 nM) or SAA+S100A12. Results from a single experiment, representative of three, are shown. (**D**) Chemical cross-linking did not identify S100A12-SAA complexes. S100A12 (1 µg) was cross-linked with SAA (1 µg) using bis[sulfosuccinimidyl] suberate (5 µM) in the presence or absence of 1 mM Ca^2+^. The gel shown is representative of two separate experiments. Gel from the same experiment was cropped to highlight relevant cross-linked treatments for the study. BS_3_, bis[sulfosuccinimidyl] suberate.

IL-8 induced with 1 nM SAA was significantly reduced by a 100-fold lower concentration of S100A12; further reduction was slight, with concentrations up to 5 nM S100A12 (Fig. 1B). When THP-1 cells were pre-incubated with SAA or with S100A12, then with S100A12 or SAA respectively, IL-8 levels produced by SAA pre-treated cells were the same as those produced following S100A12 pre-treatment (Fig. 4A), or with both agents combined (**p<0.01; ***p<0.005 compared to SAA-stimulated THP-1 cells). Taken together, these results suggest that receptor competition was unlikely.

To determine whether S100A12 altered mRNA stability, half-lives of IL-6 and TNF-α mRNA in THP-1 cells were assessed by adding actinomycin D 4 h post-stimulation and measuring mRNA levels over 3 h. Although S100A12 reduced the half-life of TNF-α mRNA in SAA-treated cells by ∼44% after 60 min, mRNA levels in SAA- and in SAA+S100A12-treated cells both reached baseline after 90 min (Fig. 4B left panel); the half-life of IL-6 mRNA was unaffected (Fig. 4B right panel).

Because mRNA levels of cytokines in cells treated with SAA+S100A12 tended to be less than those induced by SAA, whereas cytokines in supernatants were consistently and significantly reduced, we next assessed whether S100A12 blocked the secretory pathway. As predicted, the protein secretion blocker brefeldin A caused accumulation of IL-8 in SAA-stimulated THP-1 cells (Fig. 4C left panel), whereas no IL-8 accumulation was seen with SAA+S100A12 (Fig. 4C right panel), indicating reduced secretion as an unlikely mechanism.

To determine whether S100A12 complexed with SAA to modulate signaling, chemical cross-linking experiments were performed in the presence of Ca^2+^ because S100A12 was in the Ca^2+^-bound form in the culture medium used to assess its inhibitory effects. Fig. 4D shows that S100A12 migrated in SDS-PAGE gels primarily as monomer (∼10 kDa), with traces of a 15 kDa component, possibly due to conformational changes that occur when S100A12 binds Ca^2+^. Following cross-linking, S100A12 was predominantly dimeric, with low levels of tetramer (∼40 kDa). SAA has a mass of 13.5 kDa (average mass calculated from ExPASy Bioinformatics Resource Portal) and major components migrated at 13 and 15 kDa; several higher mass components were obvious. However, following cross-linking, multimeric forms (13, 26, 37, 50, 63 kDa) were separated, indicating that SAA has a marked propensity to form multivalent complexes. However, no change in the migration patterns of S100A12 or SAA were obvious following their cross-linking (Fig. 4D), indicating that they were unlikely to form complexes that may alter SAA signaling.

Suppression by S100A12 was not due to cell death. Using an Annexin V-apoptotic detection assay, we found that THP-1 cells stimulated with SAA, S100A12 or both for 15 h did not undergo early- or late-stage apoptosis (not shown).

### S100A12 Suppression was ERK1/2, but not NFκB-dependent

**Figure 5:**
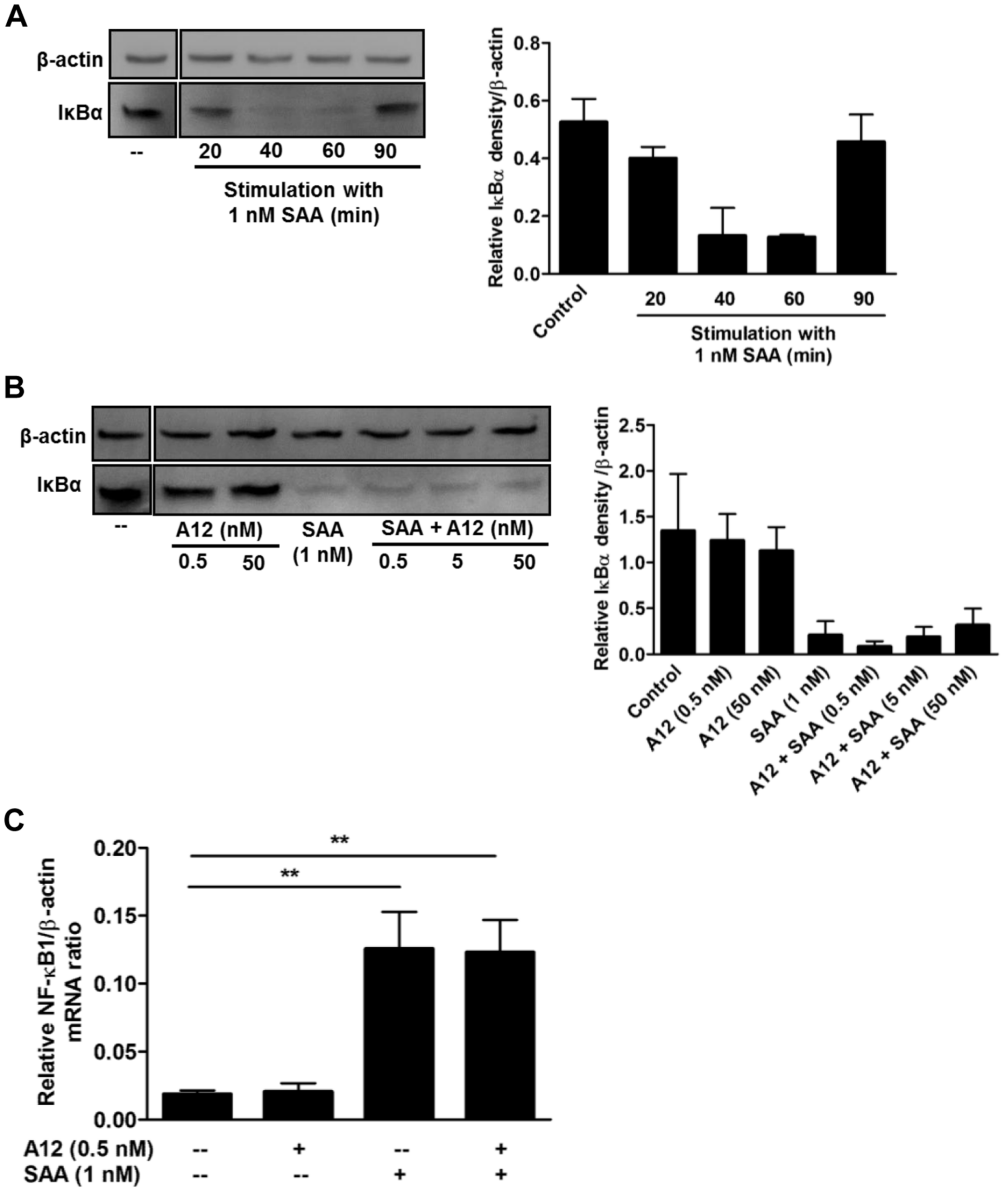
S100A12 did not alter SAA-induced IκBα degradation or NFκB1 gene expression. Western blots of cell lysates (30 µg total protein) with anti-IκBα Ab. (**A**) Time-course of SAA-induced IκBα degradation. THP-1 cells treated with SAA (1 nM) for 20–90 min. (**B**) THP-1 cells treated with S100A12 (0.5 nM or 50 nM), SAA (1 nM) or SAA (1 nM)+S100A12 (0.5 nM, 5 or 50 nM) for 40 min. Anti-β-actin Ab was used as a control for protein loading. Western blots in (**A**) and (**B**) were cropped to highlight relevant treatments for the study. (**A** and **B, right panels**) Intensities of bands corresponding to IκBα (39 kDa) were quantified by densitometry, and expressed as means ± SEM from two separate experiments. (**C**) THP-1 cells were treated with SAA (1 nM), S100A12 (0.5 nM) or SAA+S100A12 for 120 min, and NF-κB1 mRNA quantitated relative to β-actin mRNA; means ± SEM from four separate experiments; **p<0.01 compared to unstimulated control (analyzed by one-way ANOVA with Bonferroni’s correction for multiple comparison tests).

Cytokine induction by SAA in PBMC involves NF-κB activation [15]. To test whether S100A12 altered this, a time course of IκB degradation in THP-1 cells stimulated with SAA (1 nM) was established. Western blotting showed marked IκBα degradation 40 and 60 min post SAA-stimulation (Fig. 5A). No significant differences in IκBα levels were seen with 0.5, 5 or 50 nM S100A12 when compared to SAA alone (Fig. 5B). Although RAGE ligation promotes NF-κB signaling [47], and S100A12 is a putative RAGE ligand [47], S100A12 alone did not alter IκBα levels. SAA also induces NF-κB1 mRNA, which is optimal at 2 h [15]. Fig. 5C confirms NF-κB1 mRNA upregulation by SAA (**p<0.01 compared to unstimulated cells) but S100A12 had no effect on its induction. In keeping with these results, immunofluorescence studies examining NF-κB nuclear translocation by SAA ± S100A12-treated cells showed no significant differences (not shown). Together, these results indicate that S100A12 did not directly affect NF-κB signaling and downregulation of SAA-induced cytokine responses by S100A12 was likely NF-κB-independent.

**Figure 6:**
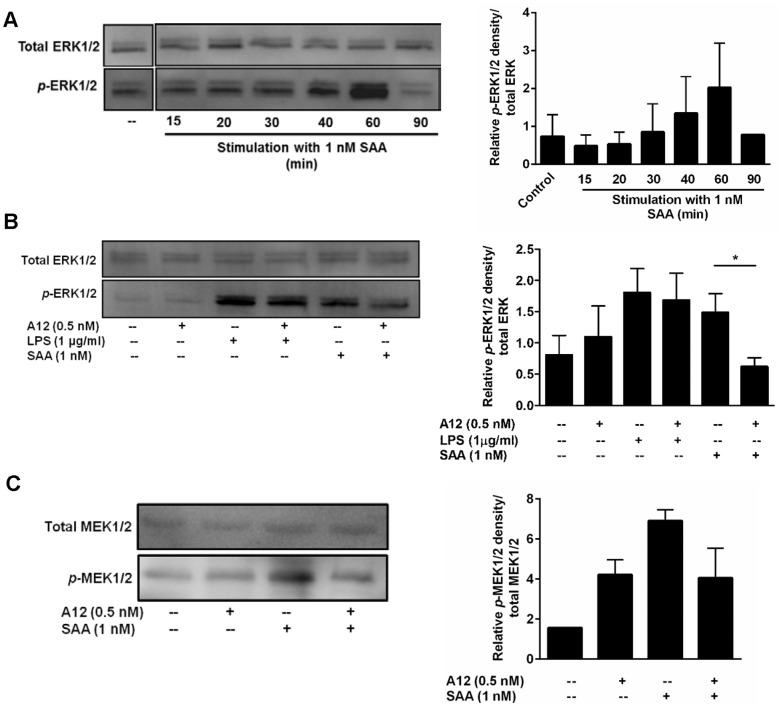
S100A12 decreased SAA-induced ERK1/2 and MEK1/2 phosphorylation. (**A**) Time course of SAA-induced *p*-ERK1/2 in THP-1 cells treated with SAA (1 nM) for the times indicated. (**B**) THP-1 cells treated with SAA (1 nM) ± S100A12 (0.5 nM) or LPS (1 µg/ml) ± S100A12 (0.5 nM) for 60 min. Western blotting performed with Ab against phospho-p44/42 (ERK1/2) MAPK. Anti-p44/42 (ERK1/2) MAPK Ab was used as a control for protein loading. Western blots shown are representative of (**A**) two or (**B**) three separate experiments. (**A** and **B, right panels**) Intensities of bands corresponding to *p*-ERK1/2 (44 and 42 kDa) were quantified by densitometry, and expressed as means ± SEM from two or three separate experiments, respectively; *p<0.05 compared to SAA alone (analyzed by one-way ANOVA with Bonferroni’s correction for multiple comparison tests). Western blots in (**A**) from the same experiment were cropped to highlight relevant treatments for the study. (**C**) THP-1 cells treated with SAA (1 nM) ± S100A12 (0.5 nM) for 60 min. Western blotting performed with Ab against phospho-MEK1/2 (Ser_217/221_). Anti-MEK1/2 Ab was used as a control for protein loading. Western blot shown is representative of two separate experiments. **(C, right panel)** Intensities of bands corresponding to *p*-MEK1/2 (45 kDa) were quantified by densitometry, and expressed as means ± SEM from two separate experiments.

**Figure 7:**
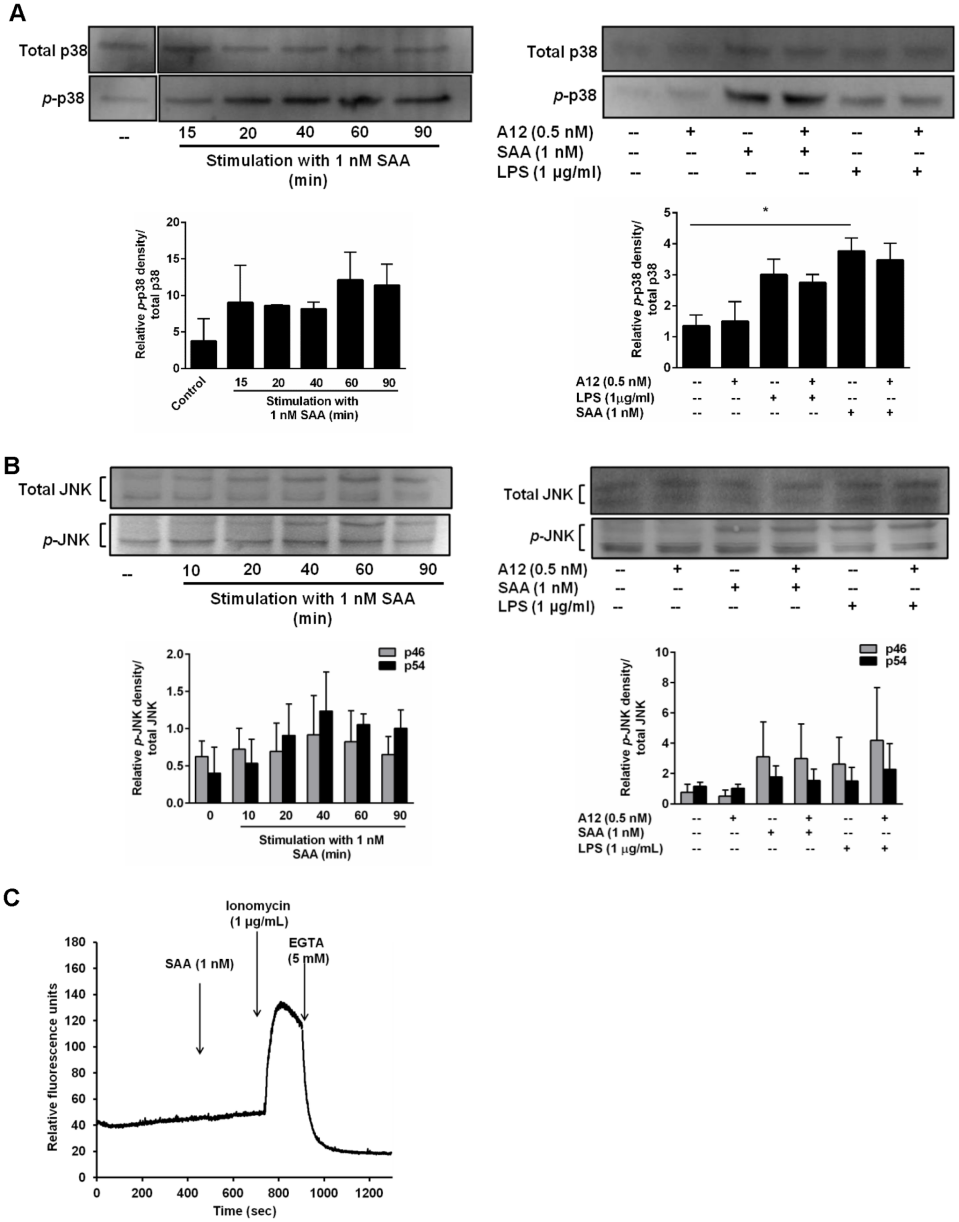
S100A12 did not alter p38 or JNK phosphorylation or provoke an intracellular Ca^2+^ flux. (**A** and **B, left panels**) Time course of SAA-induced *p*-p38 (43 kDa) and *p*-JNK (46 and 54 kDa) in THP-1 cells treated with SAA (1 nM) for the times indicated. (**A** and **B, right panels**) THP-1 cells treated with SAA (1 nM) ± S100A12 (0.5 nM) or LPS (1 µg/ml) ± S100A12 (0.5 nM) for 60 min. Western blotting performed with Ab against *p*-p38 (Thr180/Tyr182) MAPK and active JNK. Anti-p38 (Thr180/Tyr182) MAPK and anti-SAPK/JNK Ab were used as controls for protein loading. Western blots shown are representative of two or three separate experiments. Blots in (**A**) were cropped to highlight relevant treatments for the study. (**A** and **B, bottom panels**) Intensities of bands corresponding to *p*-p38 (43 kDa) and *p*-JNK (46 and 54 kDa) were quantified by densitometry, and expressed as means ± SEM from two or three separate experiments, respectively; *p<0.05 compared to unstimulated cells (analyzed by one-way ANOVA with Bonferroni’s correction for multiple comparison tests). (**C**) THP-1 cells pre-incubated with Fluo-3 acetoxymethyl ester for 1 h were stimulated with SAA (1 nM) and [Ca^2+^]_i_ measured. Ionomycin (1 µg/ml) was positive control; EGTA (5 mM), a Ca^2+^ chelator (negative control) was added to confirm Ca^2+^ involvement. Results from a single experiment, representative of two separate experiments, are shown.

SAA induction of cytokines can involve the ERK1/2 and p38 MAPK pathways [15], [16]. A time-course of ERK1/2 phosphorylation confirmed phosphorylation (primarily of ERK2), peaking 60 min after SAA stimulation (Fig. 6A). At 60 min, S100A12 did not alter basal ERK1/2 phosphorylation levels but reduced SAA-induced ERK1/2 phosphorylation almost to baseline (Fig. 6B; *p<0.05 compared to SAA-treated THP-1 cells). Consistent with this, S100A12 also reduced MEK1/2 (the principal ERK kinases) induced by SAA by ∼41% (Fig. 6C). In contrast, and in keeping with our observation that S100A12 did not alter LPS-induced IL-8 production, ERK1/2 phosphorylation triggered by LPS was unaffected by S100A12 (Fig. 6B). Although SAA promoted p38 phosphorylation that was optimal 20–60 min post-stimulation (obvious at ∼20 min, and peaking at ∼60 min), S100A12 did not reduce this, or the increase seen with LPS (Fig. 7A). Similarly, at 60 min, JNK phosphorylation increased with SAA but was not altered by S100A12 (Fig. 7B).

SAA provokes a Ca^2+^ flux in neutrophils via ligation of FPRL [28]. The Ca^2+^ signaling pathway, in particular CaM and CaMKII, is involved in some pro-inflammatory responses of activated human monocytes [70] and CaMKII can be activated in response to Ca^2+^ influx. Because ERK1/2 phosphorylation may occur subsequent to Ca^2+^ mobilization, we tested this pathway. We found no increase in Ca^2+^ influx in THP-1 cells challenged with 1 nM SAA (Fig. 7C), the concentration for optimal cytokine induction, and SAA did not promote CaMKII phosphorylation when tested over a time course of 10–40 min (not shown).

**Figure 8:**
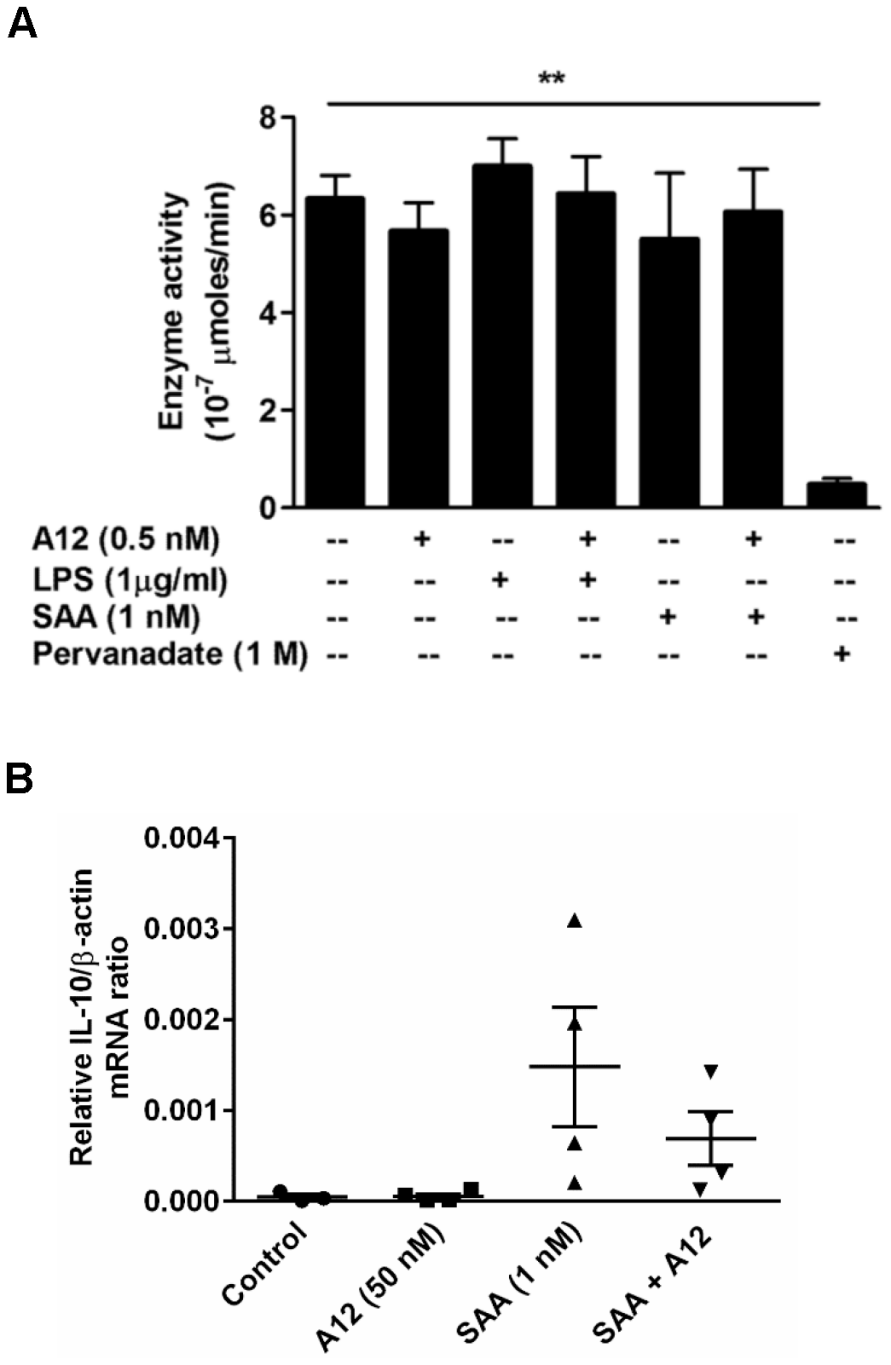
S100A12 did not alter total phosphatase activity or IL-10 mRNA induction. (**A**) pNPP phosphatase assay using THP-1 cells lysates treated with SAA (1 nM) ± S100A12 (0.5 nM), or LPS (1 µg/ml) ± S100A12 (0.5 nM) was performed. Pervanadate (1 M)-treated THP-1 cell lysates were included as positive control. Values represent means ± SEM of duplicate assays from three independent experiments; **p<0.01 compared to untreated cells (analyzed by one-way ANOVA with Bonferroni’s correction for multiple comparison tests). (**B**) PBMC treated with SAA (1 nM), S100A12 (50 nM) or SAA ± S100A12 for 4 h, and IL-10 mRNA quantitated by real-time RT-PCR; β-actin mRNA served as endogenous control. Data represent means (relative to β-actin mRNA) ± SEM of duplicate measurements from four independent donors (analyzed by paired t-test).

Because S100A12 decreased SAA-induced ERK1/2 phosphorylation, we next examined whether increased phosphatase recruitment was involved. The pNPP phosphatase assay measures activity of most phosphatases but failed to detect significant differences in levels in THP-1 cells treated with SAA or S100A12 alone, or with SAA+S100A12, whereas treatment with the universal phosphatase inhibitor, sodium pervanadate, significantly decreased activity (**p<0.01 compared to unstimulated cells) (Fig. 8A). Suppression of cytokine induction by S100A12 in SAA-stimulated PBMC could possibly be caused by increased levels of the anti-inflammatory cytokine IL-10. Consistent with the report by Lee *et al.* [71], we found that SAA increased IL-10 mRNA 4 h post-treatment, but levels were somewhat reduced by S100A12 (Fig. 8B), suggesting that suppression of pro-inflammatory cytokine production by S100A12 was unlikely due to increased IL-10.

**Figure 9:**
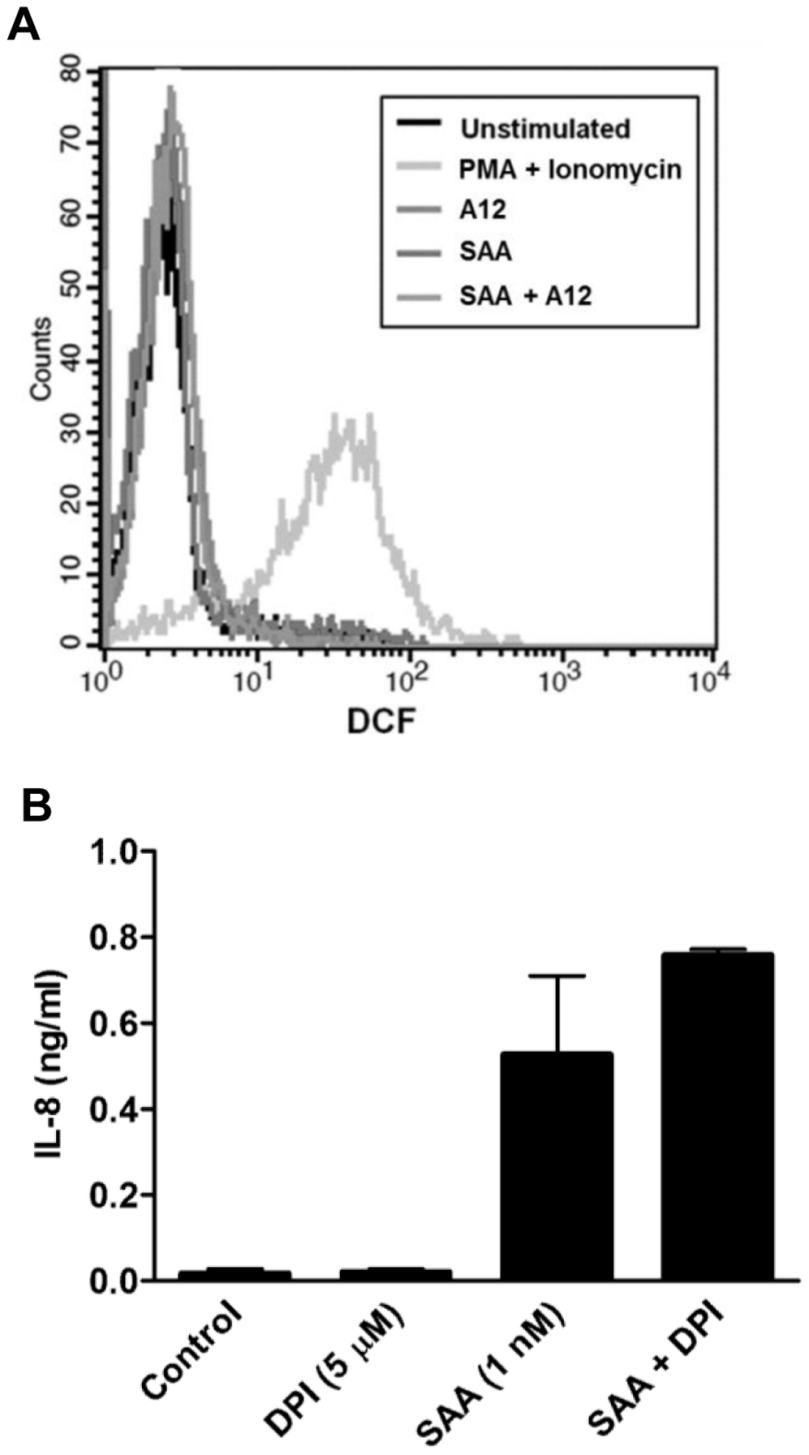
SAA at 1 nM had no effect on intracellular ROS levels. (**A**) Histogram from flow cytometric analysis of intracellular ROS levels in PBMC. The black line represents unstimulated cells; the different grey line profiles represent intracellular ROS levels, following treatments. PBMC were treated with SAA (1 nM), S100A12 (50 nM) or SAA+S100A12 for 30 min before ROS measurements. PMA+Ionomycin (10 µM) was included as positive control. Results from a single experiment, representative of two, are shown. (**B**) THP-1 cells were treated with DPI (5 µM), SAA (1 nM) or SAA+DPI, and levels of IL-8 in supernatants quantitated 15 h post-stimulation. Values are means ± SEM of duplicate assays from two separate experiments.

SAA increased ROS in SAA-treated human coronary artery endothelial cells [17], and activated NADPH oxidase in neutrophils [51]. Because intracellular ROS can mediate signaling, we investigated whether S100A12 altered intracellular ROS levels. SAA, S100A12 or SAA+S100A12 did not promote significant changes in fluorescence intensity of DCF compared to the positive control (10 µM PMA+ionomycin) (Fig. 9A). Moreover, DPI (an NADPH oxidase inhibitor; 5 µM) failed to reduce IL-8 production by SAA-treated PBMC indicating that ROS is unlikely to contribute to SAA signaling (Fig. 9B). Because SAA decreased SOD activity in human coronary artery endothelial cells [17], which may result in increased ROS, we examined effects of S100A12 by assessing inhibition of pyrogallol auto-oxidation as a function of total SOD activity but no significant changes in THP-1 cells stimulated with SAA, S100A12 or both, were seen (not shown).

## Discussion

SAA is significantly elevated in serum from patients with cardiovascular disease [7], [72] and SAA enrichment of HDL reduces its anti-inflammatory properties [73]. Moreover, overexpression of SAA by intravenous lentiviral transfer accelerates progression of atherosclerosis in ApoE^−/−^ mice, promoted by induction of chemokines and cytokines, and vascular changes that cause monocyte infiltration into lesions [14]. Importantly, localized production of SAA is implicated in the pathogenesis of inflammatory disease processes, such as recently reported in Wallerian degeneration after peripheral nerve injury [74]. Furthermore, SAA was detected in proximity to airways and in bronchoalveolar lavage fluid from patients with chronic obstructive pulmonary disease. Human lung macrophages co-localized with SAA, and SAA levels correlated with IL-8 and neutrophil elastase in bronchoalveolar lavage fluid. Consistent with effects on neutrophil recruitment and activation, SAA administered to murine lung provoked a neutrophilic response and induced chemokine (C-X-C motif) ligand-1 and -2 [26]. SAA is also elevated in the lungs of patients with severe allergic asthma. Ather *et al.* showed that SAA activates TLR-2-, MyD88-, and the NLRP3 inflammasome in the lung to provoke IL-1-dependent neutrophilic inflammation, and sensitizes mice to a mixed Th2/Th17 allergic airway disease via an IL-1R-dependent mechanism [75]. Thus, localized production of SAA in the absence of high amounts of plasma components promotes inflammation, and identification of agents that modulate this function is important.

Most studies relating to S100A12 are clinical reports showing its elevation in particular diseases, and surprisingly little is known regarding its functions. Like SAA, S100A12 was proposed to be a putative RAGE ligand that triggers activation and pro-inflammatory mediator production [47]. Others suggest its binding to N-glycans on membrane proteins, including on RAGE [76], or interaction with scavenger receptors [77]. However, we found no induction of cytokines by S100A12-stimulated mononuclear cells [41] and this was validated here. We showed that low concentrations of S100A12 are chemotactic for monocytes and mast cells independent of RAGE [49]. It is expressed by macrophages and eosinophils in human asthmatic lung and elevated levels in asthmatic sputum indicate eosinophilic asthma [42]. S100A12 activates mast cells and potentiates their responses to allergen *in vitro* in a RAGE-independent manner [42]. However, mice overexpressing human S100A12 in lung SMC have reduced peribronchial and perivascular inflammation, mucus production, and eosinophilia following acute antigen challenge in asthma, indicating a protective effect [50]. In atherosclerosis, S100A12 is expressed in macrophages [41] and in vascular SMC, and may augment calcification [78]. On the other hand, S100A12 potently inhibits MMP by sequestering Zn^2+^ and evidence of Zn^2+^-bound S100A12 complexes in human atheroma suggests this function *in vivo* [41]. Together, these results imply that S100A12 is a pleiotropic modulator that, like S100B [79], may be protective in certain conditions, and more research regarding its precise functions in specific diseases is warranted, particularly as the presence of divalent cations and/or associated binding partners, and differences in microenvironments and concentrations may govern response outcomes [80].

Our initial hypothesis was that S100A12 may potentiate pro-inflammatory effects of PAMPs (eg. LPS) or DAMPs (eg. SAA), similar to the potentiating effect reported for S100A9 on LPS-provoked responses [66]. S100A12 did not directly induce cytokines or alter cytokine levels produced by LPS-activated THP-1 monocytoid cells or by PBMC. In marked contrast, IL-6, IL-8 and TNF-α mRNA and cytokine levels from SAA activated cells were markedly reduced by S100A12 (Figs. 1C, D, E, F, G and H). Although suppression was never total, as little as 0.01 nM S100A12 was effective (***p<0.005 compared to SAA-stimulated cells); higher concentrations consistently reduced cytokine levels by ∼38% (Fig. 1B) and suppression plateaued at this level. Although SAA activates the inflammasome [75], we found no IL-1β in supernates from PBMC or THP-1 cells stimulated with SAA in the absence of ATP, and S100A12 did not influence this (not shown). Although SAA induced IL-10 mRNA, S100A12 somewhat suppressed this, suggesting that S100A12-mediated inhibition was unlikely to be IL-10-mediated (Fig. 8B). The half-lives of IL-6 or TNF-α mRNA were not significantly reduced by S100A12 (Fig. 4 B), indicating unlikely effects on mRNA stability. In addition, the cytokine secretory pathway was not altered by S100A12, as intracellular IL-8 did not accumulate in THP-1 cells treated with SAA+S100A12 (Fig. 4C). SAA induces human monocyte TF [16], but in marked contrast to cytokine induction, S100A12 did not alter basal TF levels, TF mRNA or PCA induced by SAA or by LPS (Fig. 3). Consistent with our findings, Hofmann Bowman *et al.* showed that S100A12 overexpression in airway SMC activated with TNF-α and interferon-γ attenuated chemokine (C–C motif) ligand-9 and chemokine (C-X-C motif) ligand-10 production and S100A12 overexpression in these cells in murine lung dampened allergic inflammation in the airways [50] although mechanisms were not clarified.

S100A12 and SAA have several tertiary structural similarities and both form complexes with Ca^2+^ (Fig. 4D and [81], [82]), a process proposed to facilitate S100 receptor interactions [83] but suppression by S100A12 required substantially less (100-fold) than the concentration of SAA required to induce cytokines. Moreover, pre-incubation of THP-1 cells with S100A12 reduced SAA-induced IL-8 levels by ∼40% (**p<0.01 compared to SAA-treated cells); when cells were pre-incubated with SAA, or co-incubated with S100A12 and SAA together, suppression was consistently ∼45–50% (***p<0.005 compared to SAA-treated cells; Fig. 4A) making receptor competition unlikely. Another possibility was that S100A12 and SAA formed complexes (likely non-covalent, because S100A12 has no Cys residues), thereby reducing SAA-receptor interaction. However chemical cross-linking experiments failed to detect complex formation (Fig. 4D). This method, however, relies on the presence of two primary amines in the side chain of lysine residues, or the N-terminus of each polypeptide being 11.4 Å apart for the proteins to efficiently cross-link and steric hindrance may reduce efficiency of cross-linking. The N-terminal α-helix in SAA is hydrophobic [84] and the functional hinge domain of S100A12 also forms an α-helix in hydrophobic environments [49]. More detailed examination would be required to validate this, particularly as amphipathic α-helical motif-containing peptides based on binding sites identified in CD36 ligands block SAA-induced responses in CD36-overexpressing HEK293 cells [36] although the signaling pathways blocked by these and by S100A12 appear to differ (see below).

S100A8, S100A9 and S100A12 are highly structurally homologous [63] and like SAA, all are reported to bind RAGE [85]–[87], TLR-4 [66], [88], [89] and CD36 [90]. However suppression of SAA-induced IL-8 production by S100A8, S100A9 or the S100A8/S100A9 complex was not significant at any concentration tested (Fig. 2A). Murine S100A8 was shown to induce TNF-α and IL-1β from bone marrow cells via TLR-4 and S100A9 suppressed this; S100A8/S100A9 was inactive although the complex synergized with LPS [66]. However, we found no direct TF or cytokine induction in PBMC or THP-1 cells by any S100 preparation tested, or any synergy with SAA or with LPS. Our results do not support a role for TLR-4 in extracellular functions associated with S100A12, and question this pathway in activation of human monocytes by S100A8 and S100A9. Because SAA has multiple receptors on monocytoid cells [36], and because cytokine induction by SAA was never suppressed by >50%, S100A12 appears to have specifically reduced activation of a particular pathway/s.

SAA-induced cytokine production by human neutrophils [20], [21] and monocytes [15] occurs via activation of NF-κB through the ERK1/2, p38, JNK MAPKs and PI3K pathways, whereas monocyte TF induction is principally via p38, ERK1/2 MAPK and NF-κB pathways [16]. Because NF-κB is particularly important in inflammatory responses [91], we examined whether this pathway was affected by S100A12. As for human fibroblast-like synovial cells [25], SAA provoked IκBα degradation 20 min post-stimulation of THP-1 cells, which was almost complete by 40 min (Fig. 5A); S100A12 did not alter degradation (Fig. 5B). NF-κB1 mRNA is induced 30 min post-stimulation of PBMC with SAA [15]. Consistent with this, SAA increased NF-κB1 mRNA but S100A12 had no effect (Fig. 5C), and no differences in SAA-induced nuclear translocation of the p65 subunit of NF-κB were observed (not shown). Together, these data indicate that S100A12 did not alter SAA-mediated downstream NF-κB signaling.

SAA-induced ERK1/2 was reduced to basal levels by S100A12 (Fig. 6B) whereas p38 or JNK phosphorylation (Figs. 7A, B) was unaffected. SAA-induced MEK1/2 was also reduced by S100A12 (Fig. 6C). In keeping with S100A12’s inability to reduce LPS-induced IL-8, it did not alter phosphorylated ERK1/2 levels following LPS activation (Fig. 6B). SAA provokes a Ca^2+^ flux in some cells via FPLR ligation [28] and ERK1/2 phosphorylation can occur subsequent to Ca^2+^ mobilization, but we found no evidence for this pathway (Fig. 7C). S100A12 did not alter TF induction by SAA (Fig. 3), which depends mostly on p38, and to a lesser extent ERK1/2 phosphorylation [16], tight regulation of activation by SAA appeared to occur upstream of ERK1/2. Our earlier studies showed that the MEK1/2 inhibitor PD98059 inhibited both cytokine [15] and TF [16] induction by SAA, suggesting MEK1/2 involvement in induction of both. However inhibitor studies are not always specific and must be validated by additional experiments. For example, PD98059 may increase cellular AMP:ATP ratios to activate AMP-activated protein kinase [92], and can force mitochondrial ROS generation and induce ERK1/2 phosphorylation via a Raf-1-independent/MEK-dependent mechanism [93]. We show that effects of S100A12 diverged, and the pathways regulating SAA-driven production of TF and cytokines upstream of ERK1/2 require clarification.

ERK dephosphorylation by SHP-1 phosphatase (a Src-homology 2 domain-containing protein tyrosine phosphatase-1 widely distributed in hematopoietic cells [94]), did not appear to contribute (not shown), but other phosphatases, such as the dual specificity phosphatase MKP3 [95], [96] or tyrosine phosphatases PTP-SL, HePTP and STEP [97] implicated in specifically controlling ERK1 and ERK2 activities may be involved, and require further investigation. Although the total phosphatase activity was similar in SAA ± S100A12-treated cells (Fig. 8A), a low abundance phosphatase cannot be ruled out.

SAA promotes ROS production in some cells, and intracellular ROS is implicated in MAPK activation in response to various stimuli [98]. ROS may also inactivate regulatory phosphatases, subsequently promoting ERK1/2 activation [98], [99], and antioxidants can inhibit phosphorylation [100]. However, we found no increase in basal intracellular ROS levels in PBMC (Fig. 9A), or in reduced amounts of the antioxidant SOD in THP-1 cells treated with SAA ± S100A12 (not shown). Differences seen by others could be cell-type and/or concentration-dependent. Only high SAA concentrations (10–100 µg/mL) increased ROS in neutrophils [101], and in coronary artery rings and endothelial cells [17]. In our study, 1 nM SAA (equivalent to 10 ng/mL) induced cytokines but the NADPH oxidase inhibitor, DPI had no effect on SAA-induced responses (Fig. 9B), indicating ROS-independent signaling.

Numerous receptors and multiple signaling cascades are implicated in SAA signaling and all involve MAPK activation (summarized in [36]). S100A12 specifically downregulated cytokine production, but not TF induced by SAA, by suppressing ERK1/2 phosphorylation. Interestingly, S100A12 did not reduce levels of phosphorylated ERK1/2 generated by LPS stimulation. Reminiscent of our results, cytokine induction in macrophages from CD36^−/−^ mice activated with SAA is ∼50% less than wild-type cells and ERK1/2 and JNK phosphorylation is reduced [36], although we found that JNK phosphorylation was unaffected by S100A12. We suggest that S100A12 may modulate sterile inflammation to blunt pro-inflammatory properties of lipid-poor SAA deposited in lesions where both proteins are elevated as a consequence of macrophage activation. A deeper understanding of S100A12’s receptors and/or associated binding partners within a particular inflammatory milieu is required to explain some of the incongruent functions proposed for this protein.
